# OMICS Analyses Unraveling Related Gene and Protein-Driven Molecular Mechanisms Underlying PACAP 38-Induced Neurite Outgrowth in PC12 Cells

**DOI:** 10.3390/ijms24044169

**Published:** 2023-02-20

**Authors:** Junko Shibato, Fumiko Takenoya, Michio Yamashita, Ravi Gupta, Cheol Woo Min, Sun Tae Kim, Ai Kimura, Ichiro Takasaki, Motohide Hori, Seiji Shioda, Randeep Rakwal

**Affiliations:** 1 Department of Functional Morphology, Shonan University of Medical Sciences, 16-48 Kamishinano, Totsuka-ku, Yokohama 244-0806, Japan; 2 Department of Sport Sciences, Hoshi University School of Pharmacy and Pharmaceutical Sciences, 2-4-41 Ebara, Shinagawa-ku, Tokyo 142-8501, Japan; 3 College of General Education, Kookmin University, Seoul 02707, Republic of Korea; 4 Department of Plant Bioscience, Life and Industry Convergence Research Institute, Pusan National University, Miryang 50463, Republic of Korea; 5 Department of Pharmacology, Graduate School of Science and Engineering, University of Toyama, 3190 Gofuku, Toyama 930-8555, Japan; 6 Department of Oral Biology, Graduate School of Dentistry, Tohoku University, Sendai 980-8575, Japan; 7 Institute of Health and Sport Sciences, University of Tsukuba, 1-1-1 Tennodai, Tsukuba 305-8574, Japan

**Keywords:** PACAP38, CRMP2, PC12, neuronal differentiation, genome, proteome, LC-MS/MS, DNA microarray, bioinformatics

## Abstract

The study aimed to understand mechanism/s of neuronal outgrowth in the rat adrenal-derived pheochromocytoma cell line (PC12) under pituitary adenylate cyclase-activating polypeptide (PACAP) treatment. Neurite projection elongation was suggested to be mediated via Pac1 receptor-mediated dephosphorylation of CRMP2, where GSK-3β, CDK5, and Rho/ROCK dephosphorylated CRMP2 within 3 h after addition of PACAP, but the dephosphorylation of CRMP2 by PACAP remained unclear. Thus, we attempted to identify the early factors in PACAP-induced neurite projection elongation via omics-based transcriptomic (whole genome DNA microarray) and proteomic (TMT-labeled liquid chromatography-tandem mass spectrometry) analyses of gene and protein expression profiles from 5–120 min after PACAP addition. The results revealed a number of key regulators involved in neurite outgrowth, including known ones, called ‘Initial Early Factors’, e.g., genes *Inhba*, *Fst*, *Nr4a1,2,3*, *FAT4*, *Axin2*, and proteins Mis12, Cdk13, Bcl91, CDC42, including categories of ‘serotonergic synapse, neuropeptide and neurogenesis, and axon guidance’. cAMP signaling and PI3K-Akt signaling pathways and a calcium signaling pathway might be involved in CRMP2 dephosphorylation. Cross-referencing previous research, we tried to map these molecular components onto potential pathways, and we may provide important new information on molecular mechanisms of neuronal differentiation induced by PACAP. Gene and protein expression data are publicly available at NCBI GSE223333 and ProteomeXchange, identifier PXD039992.

## 1. Introduction

Pituitary adenylate cyclase-activating polypeptide (PACAP) of the vasoactive intestinal polypeptide (VIP)/glucagon/secretin family as 27 or 38 amino acid residue (PACAP27 or PACAP38) has increasingly diverse roles and a range of biological functions [1,2,3]. PACAP bioactivity is multifunctional, acting as a neurotransmitter, neuromodulator, hypophysiotropic hormone, neuroprotector, regulator, and hormone, and it was recently reviewed in [4]. PACAP is involved in the differentiation and survival maintenance of nerve cells and the activation of neurosecretory system in the central and peripheral nervous systems, and it regulates nerve synaptic plasticity, differentiation of neural precursor cells, and glucose-dependent insulin secretion. Additionally, PACAP has a promoting effect, a cell death inhibitory effect during cerebral ischemia, and a cytoprotective effect [4,5,6]. PACAP, similar to the VIP family members, shares two common G protein-coupled receptors (GPCRs), VPAC1 R and VPAC2 R, which recognize it at the cell membrane. PACAP is believed to have a particular affinity for PAC1-R [7,8].

Studying PACAP and clarifying its function by examining a myriad of downstream (of its receptor) primary and secondary signaling pathways and networks-linkages may lead to the confirmation of the role of PACAP in numerous cellular processes, as well as contribute to the development of therapeutic drugs for diseases and disorders [5,6]. We have been studying PACAP38 role in neuroprotection, in particular, cerebral ischemia, using a mouse model of permanent middle cerebral artery occlusion (PMCAO) that resulted in the identification of a collapsing response mediator protein 2 (CRMP2) from the ischemic brain tissue post-intraventricular administration of PACAP38 [9,10] and numerous gene candidates by establishing a genome-wide approach [11]. Building on previous work, we switched to the rat adrenal-derived pheochromocytoma cell line (PC12) to further study neurite outgrowth and PACAP functionality, in relation to CRMP2 and the neuroprotective mechanisms therein [12].

Using RT-PCR, two-dimensional gel electrophoresis, in conjunction with Western blotting and inhibitor experiments, it was shown that neurite protrusion elongation by PACAP38 (10^−7^ M) in PC12 cells is mediated through the PAC1-R receptor, as demonstrated by its suppression by a specific inhibitor PA-8 [12]. These studies suggested that CRMP2 may be involved in the neurite projection elongation effect of PACAP, and the molecular mechanism was investigated in an in vitro inhibitor experiment using PC12 cells. The results showed that the projection elongation effect of PACAP is mediated by Pac1 receptor-mediated PI3K/AKT and MEK/ERK as the main pathways. Furthermore, the dephosphorylation of CRMP2 by PACAP was shown to be important for projection elongation, and since it has been reported that CRMP2 becomes inactive by phosphorylation of Thr-514, Ser-522, and Thr-555 sites by GSK-3β, CDK5, and Rho/ROCK [13,14,15,16], we investigated neurite projection elongation by PACAP and dephosphorylation of CRMP2. The results showed that the MEK/ERK inhibitor U0126 strongly suppressed projection elongation by PACAP, with no dephosphorylation of CRMP2. Thus, it is possible that MEK/ERK regulates GSK-3β, ROCK, and CDK5.

However, the inhibition of GSK-3β and ROCK resulted in Thr-514 and Thr-555 dephosphorylation by PACAP and enhanced neurite projection elongation, whereas CDK5 inhibition suppressed the projection elongation, increased CRMP2 phosphorylation, and no dephosphorylation was observed, despite CDK5 inhibition. Therefore, Ser-522 dephosphorylation by the CDK5 pathway needs to be examined, and the mechanism of neuronal differentiation by PACAP, including CRMP2 dephosphorylation, is still unclear. In our experiments, CRMP2 dephosphorylation was detected within 3 h of PACAP addition.

**Figure 1:**
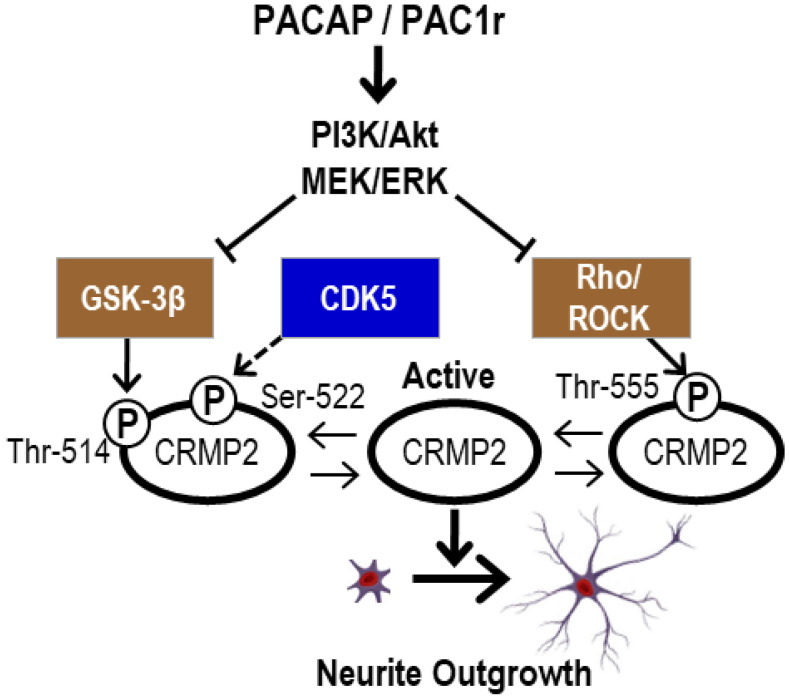
Scheme of Pac1r-mediated projection elongation of PACAP. The projection elongation action of PACAP depends on the dephosphorylation of CRMP2 through the regulation of Gsk3b, CDK5, and Rho/ROCK, with Pac1 receptor-mediated PI3K/AKT and MEK/ERK as the main pathways. The dephosphorylated CRMP2 becomes active and promotes projection elongation.

Although the pharmacological inhibition of Cdk5 with pluvaranol has been reported to result in only stepwise and incomplete dephosphorylation of CRMP2 Ser-522, and as the Cdk5 site is relatively resistant to phosphatase treatment [17], an incomplete Cdk5 inhibition would not result in increased CRMP2 phosphorylation under PACAP-added conditions; thereby, with further inhibition of projection elongation under PACAP. Therefore, it is questionable whether CDK5 is repressed by the MEK/ERK pathway to dephosphorylate CRMP2 Ser-522, as indicated by the dotted line in (Figure 1). At present, we can only suggest the indicated interactions and links on possible mechanisms by which CDK5 inhibition may regulate GSK-3β and ROCK (Figure 1).

With this background, we considered it important to identify the initial early factors involved in the neurite projection elongation effect of PACAP. Previous studies have shown that CRMP2 phosphorylation is involved in axon guidance and growth cones [18,19]. Therefore, in this report, we performed omics, especially whole genome DNA microarray (Agilent platform) and tandem mass tag (TMT)-based liquid chromatography-tandem mass spectrometry proteomics analyses to elucidate the molecular mechanism of the projection elongation effect of PACAP on CRMP2 dephosphorylation in a PC12 cell model. Dephosphorylation of CRMP2 is a therapeutic target for neurodegenerative diseases, such as Alzheimer’s disease and multiple sclerosis. We hope to contribute to the development of novel therapies by providing new insights into the mechanism by which PACAP promotes the inhibition of CRMP2 phosphorylation.

## 2. Results and Discussion

### 2.1. Genome and Proteome Analyses in PC12 Cells after Treatment with PACAP38

**Figure 2:**
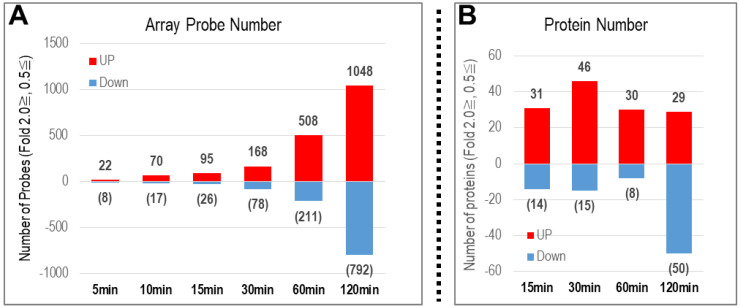
Number of genes (**A**) and proteins (**B**) whose expression was altered by PACAP in the PC12 cells. (**A**) Genes whose expression was altered by microarray analysis of PC12 cell RNA at 5, 10, 15, 30, 60, and 120 min after addition of PACAP. (**B**) Proteins with altered expression obtained by TMT-LC-MS/MS analysis of PC12 cell proteins at 15, 30, 60, and 120 min after addition of PACAP.

Two high-throughput omics techniques were utilized to investigate the gene (4 × 44K–whole rat genome DNA microarray chip) and protein (TMT-labeling of peptides, followed by LC-MS/MS) expressions in PC12 cells treated with PACAP 38 for 5, 10, 15, 30, 60, and 120 min (genomics) and 15, 30, 60, and 120 min (proteomics) (Figure 2A,B). The obtained whole-genome DNA microarray data of PC12 cells was submitted to NCBI’s GeneExpression Omnibus (https://www.ncbi.nlm.nih.gov/geo/ (accessed on 15 February 2023)) and is available under the GEO series accession number GSE223333 (https://www.ncbi.nlm.nih.gov/geo/query/acc.cgi?acc=GSE223333 (accessed on 15 February 2023)). The mass spectrometry proteomics data were deposited to the ProteomeXchange Consortium via the PRIDE [20,21] partner repository, with the dataset identifier PXD039992.

#### 2.1.1. Number of Genes with Altered Expression

Figure 2A shows the number of genes whose expression was altered by more than 2-fold and less than 0.5-fold by PACAP in PC12 cells. The number of genes, both up- and down-regulated, increased with increasing time after addition of PACAP: 22 up-regulated and 8 down-regulated genes at 5 min after the addition of PACAP, but 1048 up-regulated and 792 down-regulated genes at 120 min after the addition of PACAP.

#### 2.1.2. Number of Proteins with Altered Expression

Figure 2B shows the number of proteins whose expression was altered by PACAP by more than 2-fold and less than 0.5-fold in PC12 cells. The number of proteins increased from 29 to 46 and decreased from 8 to 50, regardless of the time course after the addition of PACAP.

##### TMT-Based Quantitative Proteomic Analysis

**Figure 3:**
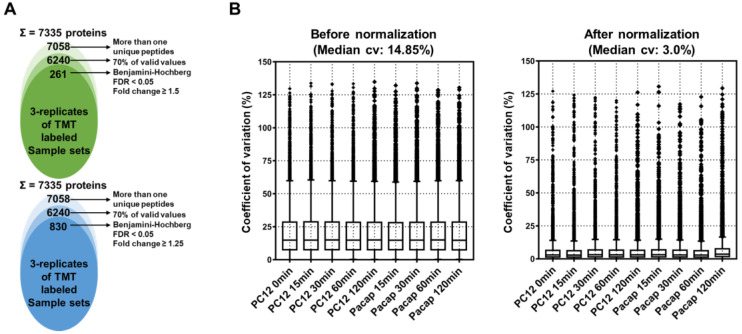
Label-free quantitative proteomic analysis of proteins extracted from PC12 cells. (**A**) Venn diagram showing the distribution of the total identified and significantly modulated proteins during different stages of analysis. The upper panel shows number of significant proteins with a fold change cutoff value of ≥1.5, while the lower panel shows significant proteins with a fold change cutoff value of ≥1.25. (**B**) Box plots showing CV values before and after the application of IRS normalization.

For the in-depth proteome analysis, TMT-labeled peptides were fractionated into 12 fractions by a basic pH-reversed phase using in-house developed stage-tips. The LC-MS/MS analysis led to the identification of 73,958 peptides and 62,854 unique peptides that matched with 7335 protein groups, with an average sequence coverage of 26.2% (Figure 3A, and Supplementary Tables S1 and S2). The removal of potential contaminants and proteins with missing values (three valid values in the three replicates at of least one group) reduced the number of identified proteins to 6240 (Figure 3A). The normalization of protein intensities across all the TMT sets was carried out by the internal reference scaling (IRS) method, which improved the coefficient of variation (CV) values from 14.85% to 3.0% (Figure 3B, and Supplementary Tables S1 and S2).

**Figure 4:**
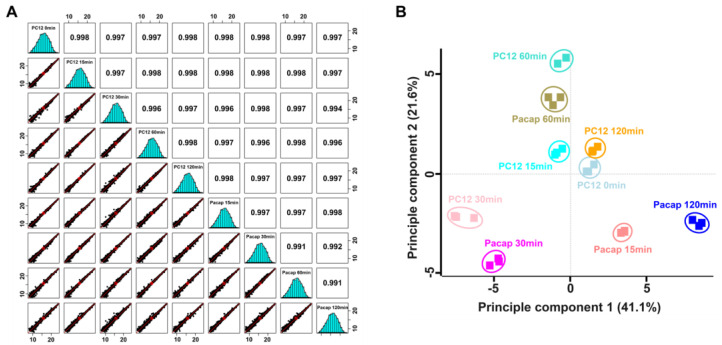
(**A**) Multi-scatter plot indicating the correlation between protein intensities among PC12 sample sets. Reproducibility across the replicate of three samples revealed by Pearson’s correlation value. (**B**) Principle component analysis of significantly modulated proteins identified by TMT-based quantitative proteome analysis.

To examine the correlation and variations among sample sets and the reproducibility of different replicates of the same sample, multi-scatter plot and principal component analyses (PCA) were performed using Perseus software. The Pearson’s correlation coefficient values of different samples were in a range of 0.991 to 0.998, indicating a high degree of correlation among different sample sets (Figure 4A). PCA plot analysis showed a clear separation of samples majorly in principle component 1, which accounted for 41.1% for the total variation (Figure 4B). Application of multiple sample test (ANOVA), controlled by a Benjamini–Hochberg FDR threshold of 0.05, led to the identification of 261 (with >1.5-fold change (FC) differences, Supplementary Table S1) and 830 (with >1.25-fold change (FC) differences, Supplementary Table S2) significantly modulated proteins.

### 2.2. Results from the DNA Microarray Analysis

#### 2.2.1. Genes Whose Expression Increased by PACAP

**Figure 5:**
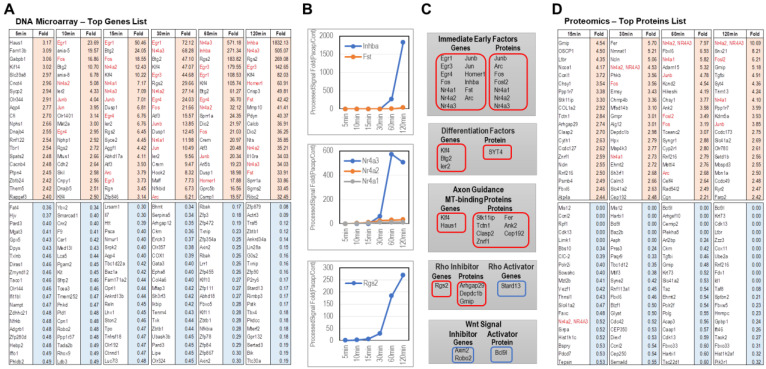
Top 20 genes and proteins whose expressions were altered by PACAP in the PC12 cells. (**A**) List of the top 20 genes whose expression was altered by DNA microarray analysis at 5, 10, 15, 30, 60, and 120 min after the addition of PACAP. (**B**) Expression changes of Inhba, Fst, NR4A1,2,3, and Rgs2 genes. Gene expression levels (ProcessedSignal) on the vertical axis are expressed as Fold (PACAP treatment/Control) values. (**C**) Top 20 genes and proteins with altered expression by PACAP, categorized as Initial Early Factors, Differentiation factors, Axon guidance, MT-binding proteins, Rho inhibitor, and genes and proteins categorized as Initial Early Factors are shown in red (up-regulated) and blue (down-regulated) boxes. (**D**) List of the top 20 proteins with altered expression obtained by TMT-LC-MS/MS analysis at 15, 30, 60, and 120 min after addition of PACAP.

The topmost up-regulated genes by PACAP were ‘Initial Early Genes’, Egr1,3,4, Fos, Nr4a1,2,3, Junb, Jun, Arc, Inhba, and Fst, including the genes known to be involved in neurite outgrowth (Figure 5A). *Inhba*: Inhba showed a 1000-fold increase in expression at 120 min after addition of PACAP, compared to no addition. Inhibin is considered to be both a growth and differentiation factor and a hormone. The homodimerization of inhibin β subunit βA is activin A (βAβA) and activin A has functions such as inducing neuronal differentiation and maintaining neuronal survival [22,23]. *Fst*: Fst (Follistatin), known to inhibit excessive differentiation by activin, was also detected. Since activin promotes strong cell proliferation, follistatin is thought to be a defense against unregulated cell proliferation and, thus, regulates cell differentiation. It is unclear how follistatin inhibits activin A, as it significantly inhibits the neurite outgrowth induced by activin A, but does not alter the neurite outgrowth induced by NGF [24]. Figure 5B graphically shows the expression changes of Inhba and Fst, and the expression levels of both Fst and Inhba increased in proportion to the passage of time by 120 min after the addition of PACAP. Fst regulates activin A even later. *Nr4a1,2,3*: The next interesting genes were Nr4A1 (Nur77), Nr4A2 (Nurr1), and Nr4A3 (NOR1), which are well-known as the orphan receptor NR4A subfamily. As for the projection elongation effect, the enhanced expression of Nur77 has been observed during NGF-induced projection elongation [25], and nur77 knockdown inhibits neurite outgrowth [26]. Similarly, the forced expression of NR4A3 has been reported to elongate neurites [27]; NR4A3 expression is upregulated by differentiation inducers, and downregulation of NR4A3 expression impairs cell differentiation [28]. Thus, Nr4A1 and NR4A3 may play an important role in the projection elongation effect of PACAP. However, as shown in Figure 5B, there is a rather large difference in the expression levels of Nr4A1 and NR4A3. Interestingly, immature cells, such as hematopoietic stem cells and pluripotent progenitor cells, express lower levels of NR4A3 than differentiated cells, and mature cells express Nr4a1 but not Nr4a3 [29]. Increase is important for projection formation, suggesting that NR4A1 may regulate the strong expression of NR4A3 even later than 120 min, such as in the relationship between Inhba and Fst described earlier. The other upregulated genes identified were Klf4, Btg2, and ler2 for neuronal differentiation, klf4 and Haus1 for axon guidance and binding, and Rgs2 for the Rho kinase inhibition.

#### 2.2.2. Genes Whose Expression Decreased by PACAP

Most of the genes whose expression was decreased were classified as transcription factors (Figure 5A). *FAT4*: FAT4 had the most decreased expression after 15 min. It is considered important for neuronal polarity and migration [30]. Genes with similar functions to FAT4 were Reln [31] and Pard3 [32]. Since the first process by which neurons acquire polarity is at the stage of axonogenesis from immature neurites, there is a need for polar proteins to be repressed during the transition from progenitor cells to normal mature neurons. *Axin2*: Axin2 is a negative regulator of the Wnt pathway that acts as a scaffolding protein for the β-catenin degradation complex [33]. Axin2 is also an important regulator of remyelination [34]. Furthermore, the activation of the Wnt/β-catenin signaling by Axin-2 knockdown in Parkinson’s disease rats reduced apoptosis, autophagy, and ROS generation, improved behavioral function, and protected nigrostriatal DA agonist neurons by increasing mitochondrial function. Upregulation of Wnt/β-catenin signaling via Axin-2 knockdown has also been reported to enhance neurogenesis of pregene (Nurr-1, Pitx-3, Ngn-2, and NeuroD1) in Parkinson’s disease rats [35]. Another was Robo2, which has been reported to inhibit Wnt signaling [36].

### 2.3. Results from the Proteome Analysis

#### 2.3.1. Proteins Whose Expression Increased by PACAP

As with the genes, many of the proteins upregulated by PACAP were proteins involved in neurite outgrowth (Egr1,3,4, Fos, Fosl2, Nr4a1,2,3, Junb, Arc) related to the ‘Initial Early Genes’ (Figure 5D). Gmip: Local inactivation of RhoA via Gmip may be involved in the regulation of dendritic development [37], and RhoA inhibitory effects have also been reported for Arhgap29 [38] and Depdc1b [39]. Fer: Downregulation of Fer expression has been reported to reduce brain damage caused by cerebral ischemia [40] and to inhibit CRMP2 phosphorylation [41]. Although the relationship with CRMP2 phosphorylation is interesting, in the present results, the amount of Fer protein peaked at 30 min after the addition of PACAP and decreased thereafter; there are reports of the formation and maintenance of intercellular adhesion as a function of Fer, and the binding of cadherins to cortical actin filaments is formation and maintenance, indicating that Fer kinase activity is important in regulating N-cadherin-mediated intercellular adhesion strength during development. N-cadherin ligation promotes tyrosine phosphorylation of Fer in wild-type fibroblasts within 15 min, reaching a peak at 30 min, followed by a significant decrease at 60 min. The decrease suggests that Fer kinase is reported to be important in the early stages of contact formation through modulation of N-cadherin mobility; the increase in N-cadherin mobility is required for the expansion of the cell contact area, which, in turn, increases adhesion strength. This expansion of cell contact area is crucial in the initiation of projection elongation, and PC12 cells that also elongate their projections show contact area expansion [42]. The increase in expression of Fer 30 min after the addition of PACAP in this study may be due to enhanced intercellular adhesion, but how CRMP2 phosphorylation by Fer affects projection elongation needs to be examined in the future. Clasp2: CLASP2 is a microtubule plus end-binding protein that promotes microtubule stabilization when overexpressed [43], and GSK3β is known to disrupt the association between CLASP2 and microtubules by phosphorylating CLASP2 [44]. It has been reported that Trim13 interacts with Nur77 and directly promotes ubiquitination and the further degradation at lysine 539 (K539), and conversely, Smad ubiquitination regulator 1 (Smurf1) can inhibit Nur77 degradation [45]. Although not shown in the data, Smurf1 was found to have decreased expression in 120 min array analysis. This suggests that NR4A1 expression may be regulated in the early stages. In addition, an increased expression of SYT4, which is involved in neuronal differentiation, as well as Stk11ip, Tctn1, Znrf1, Ank2, and Cep192 that are involved in axon guidance and binding, were observed.

#### 2.3.2. Proteins Whose Expression Decreased by PACAP

We discuss below some of the proteins in relation to neuronal differentiation (Figure 5D). Mis12: The Mis12 kinetochore complex mediates chromosome segregation in cell division. The proteins of the kinetochore form load-bearing structures that enhance the stability of bound microtubules and provide scaffolding for important signaling events during cell division. The knockdown of Mis12 in primary cultures of rat hippocampal neurons clearly altered the number of projections formed along dendrites, and the average density of filamentous pseudopodia-like projections increased by 50% upon knockdown of Mis12, suggesting its role in dendritic development [46]. Mis12 may have a function such as increasing the average density of projections by suppressing its expression in projection formation, since its expression is considerably decreased 15 min after the addition of PACAP. Cdk13: Cdk13 has been reported to be associated with Cdk5; depletion of Cdk12 or Cdk13 significantly reduces Cdk5 expression at both the mRNA and protein levels. It has also been suggested that Cdk12 and Cdk13 regulate axon elongation via a common signaling pathway that regulates Cdk5 expression [47]. Bcl9l: Bcl9l has been detected to be downregulated at 30, 60, and 120 min after the addition of PACAP. Knock-down of BCL9L reduces Wnt signaling activity. However, it has been reported that Bcl9l also acts without Wnt signaling [48]. CDC42: CDC42 is a member of the Rho family and is known to regulate neurite outgrowth with Rac1. Synaptic spine morphology is regulated by the activity of Rac1, Cdc42, and RhoA, and these finely balanced processes are crucial for the maturation process [49]. However, proteomic analysis showed that Cdc42 was reduced. This suggests that the action of Cdc42 may be suppressed in the early stages of projection formation, as it has been reported that neurons from Cdc42-deficient animals sprouted neurites, but the ability to form axons was strongly suppressed both in vivo.

### 2.4. Examination of Categories Characterized by the UP(-Regulated) and DOWN(-Regulated) in DAVID Analysis

#### 2.4.1. DNA Microarray Up-Regulated Gene Categories

HTR1A and HTR2A were categorized in the serotonergic synapse at 5 min. There are reports that neurite outgrowth is mediated via 5-HT1A and 5-HT2A receptors [50], as well as at 5 min porin, which is responsible for mediating the transport of substances into and out of the nucleus, and AQP4, in particular, has been shown to play an important role in the migration, proliferation, survival, and differentiation of adult mouse neural stem cells in vitro [51]. Other categories included ‘neuropeptide and neurogenesis’, which are important for neurite outgrowth, neurogenesis, and neuronal differentiation.

#### 2.4.2. DNA Microarray Down-Regulated Gene Categories

Axon guidance category was included in the down-genes. Receptors for the axon guidance included the semaphorin family, ephrin family, and netrin; these factors act not during the initial indistinguishable elongation of neurite projections, but later, when only one projection is elongated and it becomes axonemal in nature. These factors were considered to be suppressed at 10 min of PACAP addition because they are factors that act at the time when only one projection is elongated and becomes axonal in nature, rather than at the time of an initial indistinguishable process elongation. In addition, there are retrograde endocannabinoid signaling, glutamatergic synapse, and p53 signaling pathways, including genes that have been shown to have anti-inflammatory and neuroprotective effects when suppressed, and PTGS2 inhibition has been shown to have anti-apoptotic, anti-inflammatory, and neuroprotective effects. Among them, PTGS2 suppression has been shown to promote anti-apoptotic, anti-inflammatory, and neurite outgrowth effects [52].

#### 2.4.3. DAVID Analysis of Categories Involved in CRMP2 Dephosphorylation

**Figure 6:**
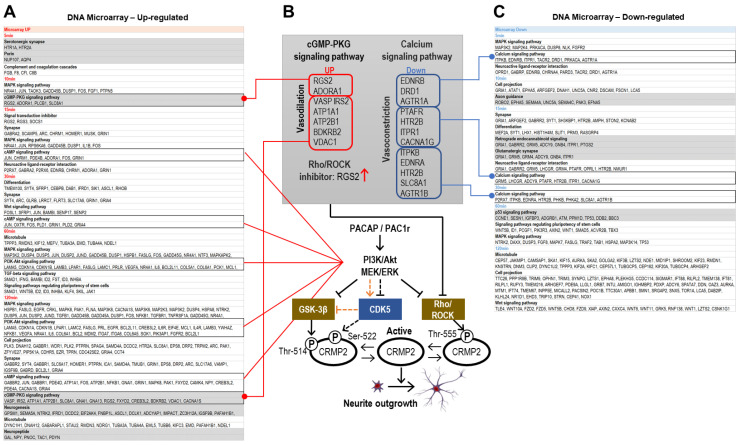
Categorical analysis using the DAVID tool of the genes (2.0-fold ≥, 0.5-fold ≤) whose expression was altered by PACAP obtained through the DNA microarray analysis. The categories mentioned in the discussion are in gray, and the categories thought to be involved in the Pac1r-mediated neurite projection elongation effect of PACAP shown in B are circled with black lines. (**A**) Categories of genes whose expression was increased by PACAP. (**B**) Genes categorized in the cGMP-PKG signaling pathway and Calcium signaling pathway that have functions of vasodilation and vasoconstriction may be involved in Rho/ROCK inhibition; red boxes, up-regulated genes; blue boxes, down-regulated genes. The red arrow indicates a strong up-regulation of the RGS2 gene. (**C**) Categories of genes whose expression was decreased by PACAP.

To investigate the dephosphorylation mechanism of CRMP2, in particular, in the neurite projection elongation pathway by PACAP, pathway analysis of genes with variable expression obtained by DNA microarray analysis was performed using the DAVID tool. The main results of the categorical analysis are shown in Figure 6A,C. The cAMP signaling pathway and the PI3K-Akt signaling pathway were detected as categories of increased gene expression (UP), and the calcium signaling pathway was detected as a (DOWN) category. cAMP signaling pathways were MEK-related. This suggests that PACAP-induced neurite projection elongation is mainly mediated by MEK/ERK and PI3K/Akt pathways. Although the categorized genes could be identified to promote vasorelaxation by attenuating Ca^2+^ signaling, many vasoconstrictor receptor genes were included. EDNRB, which was down-regulated at 5 min and down-regulated at 30 min, were the most potent vasoconstrictors. Although EDNRB is also believed to have vasodilatory effects, a recent report suggests that the up-regulation of EDNRB in vascular smooth muscle cells causes vasoconstriction [53,54]. Similarly, AGTR1A down-regulated at 5 min and AGTR1B down-regulated at 30 min are also activated by the vasoconstrictor peptide angiotensin II. PTAFR, down-regulated at 15 min, is a platelet-activating factor (PAF) receptor, and PAF not only aggregates and activates platelets and white blood cells, but also decreases blood pressure and induces biological activities, such as tracheal contraction [55].

Thus, while gene expression of many vasoconstriction-related factors is downregulated in the calcium signaling pathway, the cGMP-PKG signaling pathway in (UP)-gene category contains many vasodilation-related factors (Figure 6B). UP genes at 10 min contain the potent vasodilator adenosine receptor ADORA1, as well as the vasodilator-stimulated phosphoprotein VASP, the bradykinin receptor BDKRB2 [56], which is a hypovolemic peptide, IRS2 [57], that mediates the vasodilatory effects in perivascular fat tissue, and ATP 1A1 and ATP2B1 expression silencing leads to the development of hypertension due to vasoconstriction [58,59]. Furthermore, it has been reported that decreased VDAC1 expression may lead to cardiovascular diseases, such as pulmonary arterial hypertension [60]. RGS2 plays a role in reducing vasoconstriction and promoting relaxation by negatively regulating Gαq, which transmits signals from a variety of vasoconstrictors [61].

In the smooth muscle cells, Rho-activates Rho-kinase upon stimulation by vasoactive substances, such as noradrenaline and endothelin, results in vasoconstriction via the Rho-kinase pathway. Conversely, Rho-kinase inhibitors have been shown to suppress vasoconstriction. cGMP-PKG signaling has been reported to inhibit Gq/Rho/Rho kinase (ROCK) signaling [62]. In particular, RGS2 may be involved in the repression of Gq/Rho/Rho kinase (ROCK) signaling and is believed to promote neurite outgrowth [63]. The present DNA microarray analysis showed that RGS2 expression tended to increase up to 120 min after the addition of PACAP and was 300-fold higher at 120 min than the control (Figure 6B), suggesting that the strong increase in RGS2 expression by PACAP likely inhibits Rho-kinase, which, in turn, might be linked to CRMP2 dephosphorylation and projection elongation. In addition, we observed that many vasoconstrictors were suppressed, suggesting that this is most likely due to Rho-kinase inhibition.

## 3. Materials and Methods

### 3.1. Cell Culture

The PC12 cells (RCB0009) were obtained from the RIKEN Cell Bank (Tsukuba, Japan). Cells were cultivated in RPMI medium 1640 (ATCC Modification, Thermo Fisher Scientific, Grand Island, NY, USA) in a CO_2_ incubator (37 °C, 5%) with 5% fetal bovine serum (FBS) (16140063, Gibco, Billings, MT, USA), 10% horse serum (HS) (H1138, SIGMA, St. Louis, MO, USA), and antibiotics (penicillin-streptomycin, P4458, Sigma, St. Louis, MO, USA). Cellmatrix Type IV (Nitta Gelatin Inc., Osaka, Japan) was used to coat the culture dish. PC12 cells cultured in 10 cm sterile culture dishes were incubated for 24 h, and PACAP38 10^−7^ M was added post-incubation. PC12 cells after 5, 10, 15, 30, 60, and 120 min post-incubation from PACAP38 addition were used for total RNA extraction for DNA microarray analysis [9,10,11,12]. Similarly, PC12 cells after 15, 30, 60, and 120 min of incubation from PACAP addition were used for total protein extraction for proteomic analysis [9,10,12].

### 3.2. DNA Microarray Analysis

Total RNA was extracted from the cells using the RNeasy Mini Kit (74104, Qiagen, Germantown, MD, USA) [9,10,11]. To verify the quality of this RNA, yield and purity were determined spectrophotometrically on a DeNovix DS-11 spectrophotometer (DeNovix, Wilmington, DE, USA), and confirmed using formaldehyde-agarose gel electrophoresis. To check the quality of the synthesized cDNA using the Affinity Script QPCR cDNA synthesis kit (600559, Agilent Technologies Inc., Santa Clara, CA, USA), PCR reaction was performed to confirm the expression of house-keeping genes (beta-actin or GAPDH) using Emerald Amp PCR Master (RR300A, Takara, Japan). PCR products were separated on a 1.6% agarose gel and visualized with ethidium bromide staining under UV light. Total RNA extracted from PC12 cells (*n* = 6), pooled prior to DNA microarray analysis (Whole Rat Genome DNA Microarray 4 × 44K, G4131F; Agilent Technologies Inc., Santa Clara, CA, USA). Microarray experiment was described previously [9,10]. Total RNA (400 ng) was labeled with either Cy3 or Cy5 dye using an Agilent Low RNA Input Fluorescent Linear Amplification Kit (Agilent Technologies Inc., Santa Clara, CA, USA). Fluorescently labeled targets of control, as well as treated samples, were hybridized to the same microarray slide with 60-mer probes. A flip labeling (dye-swap or reverse labeling with Cy3 and Cy5 dyes) procedure was followed to nullify the dye bias associated with unequal incorporation of the two Cy dyes into cDNA, and to select the differentially expressed genes by the dye-swap approach. Hybridization and wash processes were performed according to the manufacturer’s instructions, and hybridized microarrays were scanned using an Agilent Microarray scanner G2505C (Agilent Technologies Inc., Santa Clara, CA, USA).

For the detection of significantly differentially expressed genes between control and treated samples, each slide image was processed by Agilent Feature Extraction software (version 9.5.3.1). This program measures Cy3 and Cy5 signal intensities of whole probes. Dye-bias tends to be signal intensity dependent; therefore, the software selected probes using a set by rank consistency filter for dye-normalization. Said normalization was performed by LOWESS (locally weighted linear regression), which calculates the log ratio of dye-normalized Cy3- and Cy5-signals, as well as the final error of log ratio. The significance (P) value based on the propagate error and universal error models. In this analysis, the threshold of significant differentially expressed genes was <0.01 (for the confidence that the feature was not differentially expressed). In addition, erroneous data generated due to artifacts were eliminated before data analysis using the software.

The functional categories (KEYWORDS) and pathway (KEGG pathway) of the list of variable genes selected by the microarray analysis were analyzed using the Database for Annotation, Visualization, and Integrated Discovery (DAVID) v6.8.

### 3.3. Proteome Analysis

For extracting the proteins, PC12 cells were isolated by the addition of LB-TT extraction solution (7 M (*w*/*v*) urea, 42 g; 2 M (*w*/*v*) thiourea, 15.2 g; 4% (*w*/*v*) CHAPS, 4.0 g; 18 mM (*w*/*v*) Tris-HCl (pH 8.0), 1.8 mL; 14 mM (*w*/*v*) Trizma base, 169.5 mg; 0.2% (*v*/*v*) Triton X-100; 0.2 mL 50 mM (*w*/*v*) DTT, 771.5 mg; 1% (*v*/*v*) pH 3–10 ampholyte, 1 mL; and two EDTA-free proteinase inhibitor (5892791001, Roche, Basel, Switzerland) tablets in a total volume of 100 mL)) to lyse the cells [9,10,12]. One (1) mL of LB-TT was quickly added to the culture dish and immediately mixed for 1 minute at room temperature. Protein concentration was determined with a Pierce™ 660 nm Protein Assay Reagent (Thermo Fisher Scientific, Waltham, MA, USA) using bovine serum albumin (BSA) as a standard and a DeNovix DS-11 spectrophotometer.

#### 3.3.1. Protein Digestion by Filter-Aided Sample Preparation (FASP)

Protein digestion was carried out using a filter-aided sample preparation (FASP) approach, as described in a previous study [64,65]. Briefly, acetone-precipitated proteins (300 μg) were dissolved in 30 μL of denaturation buffer (4% sodium dodecyl sulfate (SDS) and 100 mM dithiothreitol (DTT) in 0.1 M tetraethylammonium tetrahydroborate (TEAB), pH 8.5. After sonication of the sample for 3 min and heating at 99 °C for 30 min, denatured proteins were loaded onto a 30-kDa spin filter (Merck Millipore, Darmstadt, Germany) and diluted with UA buffer (8 M urea in 0.1 M TEAB, pH 8.5) to a final volume of 300 μL. The filter units containing proteins were washed, and the buffer was exchanged three times using 300 μL of UA buffer by centrifugation at 14,000× *g* for removal of SDS. After removing SDS from the samples, cysteine alkylation was accomplished through the addition of 200 μL of alkylation buffer (50 mM iodoacetamide (IAA), 8 M urea in 0.1 M TEAB, pH 8.5) for 1 h at room temperature in the dark. Then, the buffer was exchanged with UA buffer to TEAB buffer (50 mM TEAB, pH 8.5) in a spin filter unit. The protein was digested with trypsin (enzyme-to-substrate ratio (*w*/*w*) of 1:100) dissolved in 50 mM TEAB buffer containing 5% acetonitrile (ACN) at 37 °C overnight. After overnight digestion, the digested peptides were collected by centrifugation, and the filter device was rinsed with 50 mM TEAB and 50 mM NaCl. The peptide concentrations were measured using the Pierce Quantitative Fluorometric Peptide Assay kit (Thermo Fisher Scientific, Waltham, MA, USA), according to the manufacturer’s instructions.

#### 3.3.2. Peptides Labeling with Tandem Mass Tags (TMT), Desalting, and Basic pH Reversed Phase (BPRP) Peptide Fractionation Using Stage-Tip

TMT labeling of digest peptides was performed as described previously [65,66,67] using a TMT 10-plex kit (Thermo Fisher Scientific, Waltham, MA, USA). Briefly, each TMT reagent (0.8 mg) was dissolved in 120 μL of anhydrous ACN, of which, 25 μL was added to each sample. Prior to incubation each sample set, additional ACN was added as 30% of final concentration. After incubation at room temperature for 1 h, the reaction was quenched with hydroxylamine to a final concentration of 0.3% (*v*/*v*). After TMT-labeling, all the labelled peptides across all samples were combined and lyophilized. The pooled TMT-labeled peptides were desalted using the HLB OASIS column (Waters, Milford, MA, USA), according to the manufacturer’s instruction. After desalting, peptides were lyophilized again and dried peptides were reconstituted in 200 μL of loading solution (15 mM ammonium formate, 2% ACN) and were fractionated into 12 fractions using in-house developed stage-tips, prepared by packing C18 Empore disk membranes (3M, Bracknell, UK) at the bottom and POROS 20 R2 reversed phase resin (Thermo Fisher Scientific, Waltham, MA, USA) into 200 μL yellow tip. Prior to peptide loading, the stage-tip was washed with 100% methanol, 100% ACN and equilibrated with loading solution. The peptide was loaded, and 12 fractions were subsequently eluted with pH 10 buffer solution containing 5, 8, 11, 14, 17, 20, 23, 26, 29, 32, 35, 41, 44, 60, 80, and 100% ACN, as described previously. Finally, the 12 fractions were lyophilized in a vacuum centrifuge and stored at −80 °C until further LC-MS/MS analysis.

#### 3.3.3. Q-Exactive MS Analysis

Obtained peptides were dissolved in solvent-A (water/ACN, 98:2 *v*/*v*; 0.1% formic acid) and separated by reversed-phase chromatography using a UHPLC Dionex UltiMate^®^ 3000 (Thermo Fisher Scientific, Waltham, MA, USA) instrument [68]. For trapping the sample, the UHPLC was equipped with Acclaim PepMap 100 trap column (100 μm × 2 cm, nanoViper C18, 5 μm, 100 Å) (Thermo Fisher Scientific, Waltham, MA, USA) and subsequently washed with 98% solvent A for 6 min at a flow rate of 6 μL/minutes. The sample was continuously separated on an Acclaim PepMap 100 capillary column (75 μm × 15 cm, nanoViper C18, 3 μm, 100 Å) at a flow rate of 400 nL/minute. The LC analytical gradient was run at 2% to 35% solvent B (100% ACN and 0.1% formic acid) over 90 min, then 35% to 95% over 10 min, followed by 90% solvent B for 5 min, and finally, 5% solvent B for 15 min. Liquid chromatography-tandem mass spectrometry (LC-MS/MS) was coupled with an electrospray ionization source to the quadrupole-based mass spectrometer QExactive™ Orbitrap High-Resolution Mass Spectrometer (Thermo Fisher Scientific, Waltham, MA, USA). The resulting peptides were electro-sprayed through a coated silica emitted tip (Scientific Instrument Service, Amwell Township, NJ, USA) at an ion spray voltage of 2000 eV. The MS spectra were acquired at a resolution of 70,000 (200 *m*/*z*) in a mass range of 350–1650 *m*/*z*. The automatic gain control (AGC) target value was 3 × 10^6^ and the isolation window for MS/MS was 1.2 *m*/*z*. Eluted samples were used for MS/MS events (resolution of 35,000), measured in a data-dependent mode for the 15 most abundant peaks (Top15 method), in the high mass accuracy Orbitrap after ion activation/dissociation with higher energy C-trap dissociation (HCD) at 32 collision energy in a 100–1650 *m*/*z* mass range [68]. The AGC target value for MS/MS was 2 × 10^5^. The maximum ion injection times for the survey scan and MS/MS scan were 30 ms and 120 ms, respectively.

#### 3.3.4. Data Processing Using MaxQuant Software and Data Analysis Using Perseus and R Program

The MaxQuant software (version 1.6.5.0, Max Planck Institute of Biochemistry, Munich, Germany) was used to apply for a database search as described previously [66,69,70]. All the three technical replicates were cross-referenced against the rat (*Rattus norvegicus*) and mouse (*Mus musculus*) databases (36,180 and 86,074 entries, respectively, https://https://www.uniprot.org/ (accessed on 26 October 2019). TMT data processing of two different data was performed using default precursor mass tolerances set by the Andromeda search engine, which was set to 20 ppm for the first search and 4.5 ppm for the main search. Reporter mass tolerance has to set the minimum as 0.003 Da, and the minimum reporter precursor ion fraction (PIF) was set as 0.5. The product mass tolerance was set to 0.5 Da, and a maximum of two missed tryptic cleavage were allowed. Carbamidomethylation of cysteine residues and acetylation of lysine residues and oxidation of methionine residues were specified as fixed and variable modifications, respectively. A reverse nonsense version of the original database was generated and used to determine the FDR which was set to 1% for peptide identifications. Statistical analysis was carried out using Perseus software (ver. 1.6.17.0) (Max Planck Institute of Biochemistry, Munich, Germany) [70]. The normalization of reporter ion intensities was carried out using an internal reference scaling (IRS) method as described previously [65,71]. Briefly, we employed the multiple normalizations through Bioconductor of R program, in which, first sample loading (SL) normalization among the samples was conducted for corrections of sample loading errors using mean values calculated from sum value of each column, and IRS method was applied in second normalization among the technical replicates to normalize the differences in the intensity values that arise because of separate MS runs. Missing values imputation was carried out from a normal distribution (width: 0.3, downshift: 1.8) using Perseus software [70]. Multiple sample test, controlled by the Benjamini–Hochberg FDR threshold of 0.05, was applied to identify the significant differences in the protein abundance (≥1.25- and ≥1.5-fold change). The mass spectrometry proteomics data have been deposited to the ProteomeXchange Consortium via the PRIDE [20,21] partner repository, with the dataset identifier PXD039992.

## 4. Conclusions

This targeted omics analyses detected many molecular factors that are important in the early stage of neurite projection elongation by PACAP, which is one of the highlights of this study. Moreover, as this highly diverse bioactive peptide has roles in neurogenesis/differentiation, embryonic development, and neuronal repair, and therein brain development, these results reinforce the importance of continued research into not only fundamental research into signaling mechanisms downstream of PACAP, but also gaining hints from the omics data to provide therapeutic possibilities for brain pathologies [72], including stroke, one of the first reasons our group has been researching PACAP38 [11].

An intriguing factor was the Rgs2, a member of the regulator of G protein signaling (RGS) proteins, which is responsible for the rapid turnoff of G protein-coupled receptor signaling pathways [73]. RGS2, which is involved in Rho/ROCK inhibition, may be important in the CRMP2 dephosphorylation by PACAP. Future studies will investigate the CRMP2 dephosphorylation and projection elongation effects of RGS2; CRMP2 dephosphorylation of CDK5 could not be discussed deeply from the present results. However, proteome analysis detected Axin2, suggesting that Cdk5, as indicated by the orange (dotted) arrow in Figure 6B, may suppress the GSK-3β. Axin is phosphorylated by Cdk5, and this phosphorylation promotes the interaction between Axin and GSK-3β, and the interaction between Axin and GSK-3β was also observed. It has been reported that GSK-3β is negatively regulated by Cdk5, which is important for neuronal polarization and axonal development [74,75,76]. Therefore, it would be interesting to see whether phosphorylation of Axin2 by CDK5 leads to inhibition of GSK-3β activity, and the possibility of GSK-3β-mediated CRMP2 dephosphorylation by CDK5 would be interesting to clarify in the future.

Pac1r activation by PACAP prevents tau accumulation and improves cognitive performance in mice, indicating a potential therapeutic approach for AD and other tauopathies [77]. We are currently conducting CRMP2 dephosphorylation and omics analysis in Pac1r-KO mouse brain tissue and hope to elucidate Pac1r-mediated dephosphorylation of CRMP2 by PACAP, which will contribute to the development of early prevention methods to reduce the risk of disease development, as possible.

## Data Availability

The data presented in this study are available in the article and submitted databases. The raw data are available upon a reasonable request from the corresponding author.

## Supplementary Materials

The following supporting information can be downloaded at: https://www.mdpi.com/article/10.3390/ijms24044169/s1.
